# Positive social behaviours are induced and retained after oxytocin manipulations mimicking endogenous concentrations in a wild mammal

**DOI:** 10.1098/rspb.2017.0554

**Published:** 2017-05-24

**Authors:** Kelly J. Robinson, Sean D. Twiss, Neil Hazon, Simon Moss, Patrick P. Pomeroy

**Affiliations:** 1 Sea Mammal Research Unit, Scottish Oceans Institute, University of St Andrews, St Andrews, UK; 2 Department of Biosciences, Durham University, Durham, UK; 3 Scottish Oceans Institute, University of St Andrews, St Andrews, UK

**Keywords:** oxytocin, manipulation, social behaviour, proximity-seeking behaviour, seal, intravenous

## Abstract

The neuropeptide hormone oxytocin modulates numerous social and parental behaviours across a wide range of species, including humans. We conducted manipulation experiments on wild grey seals (*Halichoerus grypus*) to determine whether oxytocin increases proximity-seeking behaviour, which has previously been correlated with endogenous oxytocin concentrations in wild seal populations. Pairs of seals that had never met previously were given intravenous injections of 0.41 µg kg^−1^ oxytocin or saline and were observed for 1 h post-manipulation. The dose was designed to mimic endogenous oxytocin concentrations during the observation period, and is one of the lowest doses used to manipulate behaviour to date. Seals given oxytocin spent significantly more time in close proximity to each other, confirming that oxytocin causes conspecifics to seek others out and remain close to one another. Aggressive and investigative behaviours also significantly fell after oxytocin manipulations. Despite using a minimal oxytocin dose, pro-social behavioural changes unexpectedly persisted for 2 days despite rapid dose clearance from circulation post-injection. This study verifies that oxytocin promotes individuals staying together, demonstrating how the hormone can form positive feedback loops of oxytocin release following conspecific stimuli, increased motivation to remain in close proximity and additional oxytocin release from stimuli received while in close proximity.

## 1. Introduction

Behaviour is frequently perceived as the most plastic and adaptable aspect of an organism. Many types of behaviour change in response to external stimuli, whether from environmental, conspecific or heterospecific origin. However, all behaviour is ultimately constrained by the underlying physiology that governs an individual's ability to perceive and act on relevant opportunities for behavioural expression. Hormones have wide-ranging effects on behaviour as they influence it in two fundamental ways. First, neuropeptide hormones influence the brain structures and cognitive processes behind the motivation to perform behaviours. Second, hormones in circulation can act on peripheral tissues to ensure the relevant anatomical constructs are present to successfully carry out behaviours. It is therefore vital to understand the role hormones play in behaviour, and how they link stimuli perception, central processes and outward actions.

Interacting with other individuals is a fundamental part of every organism's existence. Hormones have been shown to mediate a variety of responses to other individuals, ranging from hostile [1] to affiliative [2] exchanges. Of these hormonally mediated interactions, an individual's ability to recognize another individual and adapt their behaviour towards them based on past experiences is a crucial and complex one. Instances of this can occur within or across species [3], and if interactions are repeated frequently enough, they can lead to the formation of social bonds, which can vary in importance from temporary associations between group members to parental bonds between adults and dependent offspring. Despite the variation in types of social or parental bond that exist in the natural world, many are mediated by oxytocin, or one of its related forms [4].

Oxytocin has been shown to correlate with several pro-social behaviours in a variety of animal species including humans [5]. Nevertheless, to show a causal link between hormones and behaviour, manipulation experiments must be carried out. Oxytocin manipulations have been performed with a range of captive, laboratory or domestic animals and humans (electronic supplementary material, SM1), showing that oxytocin stimulates behaviours that are crucial for social bonding between unrelated individuals, for parent–infant bonding and care giving. However, only one manipulation study has occurred to date on an animal species in its natural environment (meerkat, *Suricata suricatta* [6]), and no published study outside of laboratory settings has ever demonstrated that pro-social behaviours which correlate to elevated oxytocin concentrations naturally are definitely caused by changes in the hormone via manipulation studies. Studies of behavioural expression in natural settings are crucial as they truly embody the complex variation in conspecifics and environmental conditions organisms encounter [7]. Manipulations given to study organisms additionally must be carefully designed to ensure the natural system is replicated as closely as possible. This in itself is difficult, despite the popularity of captive manipulation studies the methodologies and doses used for such research vary widely. There is also concern that manipulation studies generate supraphysiological concentrations in trial subjects, meaning any behavioural changes caused by such manipulations may not occur in natural systems, while also potentially negatively impacting on non-target tissues such as the heart or digestive tract [8].

Published studies investigating the oxytocin system only exist for five mammalian species in the wild (meerkat [6]; chacma baboon, *Papio* *hamadryas ursinus* [9]; chimpanzee, *Pan troglodytes* [10,11]; harbour seal, *Phoca vitulina*, and grey seal, *Halichoerus* *grypus* [12–14]). Of these, most is known about oxytocin in the grey seal. The plasma clearance rate and plasma concentrations for a variety of age groups, reproductive statuses and both sexes has been documented in this species, all important requirements for developing appropriate manipulation doses [12,13]. In addition, pro-social behaviours have been correlated with high endogenous plasma oxytocin in this species (increased proximity seeking behaviour between mothers and pups [13]), and unrelated seals show reduced aggression and investigative behaviour towards individuals they have previously co-habited with [14], both pro-social behaviours that are easy to observe and record in wild individuals.

We aim to determine whether a causal link exists between elevated oxytocin concentrations and increased proximity-seeking behaviour between conspecifics, which previous correlation studies of endogenous oxytocin concentrations have suggested [13]. Additionally, we aim to demonstrate that intravenous (IV), biologically relevant oxytocin doses are capable of triggering pro-social behaviours in a wild mammal outside of laboratory conditions, confirming that peripherally administered exogenous oxytocin can reach relevant brain regions and trigger significant behavioural changes [15].

## 2. Material and methods

### (a) Study site

This study took place on the Isle of May grey seal breeding colony in Scotland (56°11′ N, 02°33′ W) from 18 November to 4 December 2011.

### (b) Pen design and construction

Pens were constructed as described by Robinson *et al*. [14,16], with permission from Scottish National Heritage and in accordance with UK Home Office guidelines on suitable temporary holding facilities for grey seals, and were taken down once the trials were complete. Holding pens measured approximately 15 × 10 m each and contained pools of fresh water. Experiments took place in a separate 5 × 5 m trial pen out of sight of the holding pens. Holding pens were located on a part of the island the breeding seals did not use. The holding pens were separated by 10 m to ensure that no interactions could occur through the fencing outside of the trials and foliage growing naturally on the Isle of May blocked line of sight between the pens.

### (c) Study animals

20 newly weaned grey seal pups were penned in two trial cohorts on the Isle of May in 2011 for the manipulation trials. Each cohort used 10 weaned pups penned in two groups of five for 8 days. The second cohort was captured immediately after the first cohort was released. Pups were defined as being weaned on the second consecutive day of being seen without their mother after a normal rearing period, based on daily observations [17]. All the study animals had weaned within 5 days of each other. Captured pups were placed into one of two holding pens to generate two groups of five individuals that had never encountered the individuals in the other pen. The sex ratio within the two cohorts was even.

Regular observations on mother–pup pairs at various sites scattered throughout the Isle of May breeding colony allowed us to be confident that individuals in one pen had not come into contact previously with the individuals in the other. After a day's rest following capture to acclimate to the holding pens, manipulation trials then commenced on the second day after capture.

Adult seals were not suitable for this study as it would be impossible to guarantee individuals had never previously met, and due to the practical difficulties associated with their large size and aggressive behaviour. Weaned pups were deemed a suitable substitute for adults as the oxytocin receptor system in brain regions associated with social behaviours are mature at weaning in rodents [18], and weaned pups are capable of pro-social behaviour at this age [14]. The use of newly weaned individuals allowed us to take advantage of the natural one- to four-week post-weaning fast in this species [19], and collection from different sites of the breeding colony ensured that individuals in the two separate pens had not encountered each other previously.

Upon capture all pups were sexed and weighed. Pups were weighed ±0.2 kg on a spring balance (Salter Industrial Measurements, West Bromwich, UK). Pups less than 30 kg initially were excluded from the study. Early release criteria based on mass loss was set in accordance with previous studies to avoid extending the post-weaning fasting period unnaturally [14,16,17]. None of the study animals lost sufficient mass to warrant early release. To assist in identification, all animals received a temporary paint mark on the mid-dorsal region. All pups were released into the colony after they participated in the study.

### (d) Plasma sampling and analysis

Plasma samples were collected from study individuals upon capture and entry into the holding pens from the extradural vein into two 10 ml lithium heparin vacutainers (Becton, Dickinson and Company), and were kept on ice until they could be spun and frozen at −20°C, as described in [14]. This sample collection and storage methodology has been shown to induce negligible oxytocin degradation over at least a 2-year period [12]. Additionally, it has been previously demonstrated that oxytocin concentrations in phocid seal plasma does not change with capture stress [12] or throughout up to two weeks of captivity [14]. Plasma samples were also collected after a subset of saline (*n* = 8) and oxytocin (*n* = 8) manipulation trials to determine whether the manipulations were successful in raising basal oxytocin concentrations for the hour-long trial or determine if the injection procedure alone elevated plasma oxytocin concentrations.

Plasma was analysed for oxytocin using an ELISA (Assay Designs, Ann Arbor, MI, USA) with each sample undergoing solid-phase extraction using Sep-Pak C18 columns prior to analysis following the methodology validated for detecting phocid plasma oxytocin [12]. After the plate was read using a BioTek ELx800 reader, the standard curve and assay results were fitted using the calibFit package [20] in R v. 2.9.2 [21]. Recovery rates for the extraction and ELISA procedure were 107.2% (*n* = 10), intra-assay coefficient of variance (COV) for this assay was 6.3% and inter-assay COV over the five plates used in this study was 13.6%.

### (e) Manipulation protocol

A dose of 0.41 µg kg^−1^ of a commercially available oxytocin solution (10 iu ml^−1^ or 0.18 mg ml^−1^; Oxytocin-S, Intervet UK) was selected for use as the IV oxytocin manipulation in this study based on previous work optimizing dosage for IV administration in phocid seals (electronic supplementary material, SM2). This dose was the lowest that was successful in elevating plasma oxytocin concentrations for one hour in a series of pilot studies (electronic supplementary material, SM2), the duration of the pen trials in this study.

IV injections of oxytocin doses were administered using manual restraint and were delivered into the extradural vein. It is not possible to efficiently administer doses intranasally to phocid seals due to their ability to close their nostrils and hold their breath for over ten minutes [22]. As peripheral injections have been shown to elicit behavioural changes in free-roaming mammals (meerkats [6]) and can be delivered rapidly to manually restrained seals (see below), IV injections were selected for use in this experiment. Saline manipulations were conducted to act as a control. The volume of sterile saline solution injected was calculated to be equal to the volume that would be injected when the individual was given an oxytocin manipulation. This was calculated by converting the 0.41 µg kg^−1^ dose into the volume of the 0.18 mg ml^−1^ oxytocin solution that would be injected, 0.0023 ml kg^−1^.

### (f) Trial protocol

Each trial occurred across 3 days.

*Day 1*: Initial meeting. One seal from each holding pen was placed in the trial pen for one hour. Both individuals were given an IV injection of either saline or oxytocin at doses described above. After the trial, participants were separated and returned to their original holding pen.

*Day 2*: Rest day.

*Day 3*: Second meeting. The same two individuals were placed in the trial pen for another hour and both were given an IV injection of whichever manipulation type they did not receive on the first day of the trial (saline/oxytocin). After the second meeting trial both individuals were returned to their original holding pens.

Whether a trial pair was given the oxytocin or saline manipulation first was randomized to investigate any order effects in giving the manipulation. The same pair of individuals was used for an oxytocin and saline control manipulation as consistent individual differences in investigative [23] and aggressive behaviour [14,16] are present in both adult and newly weaned grey seals. Newly weaned seals cannot recognize other conspecifics after only one hour of exposure to them, and treat them as if they are novel individuals [16]. They show no change in the frequency of aggressive, investigative or affiliation behaviour towards each other, and no change in the amount of time in close proximity to each other [16]. Therefore, there should be no difference in behaviours expressed across the first and second meeting other than those caused by the saline or oxytocin manipulations.

The two subjects required for a trial were captured in the holding pens simultaneously and administered whichever manipulation they were to receive for their trial that day (IV oxytocin or saline). They were then transported while restrained in a bag to the trial pen at the same time. Time spent capturing, injecting manipulations and transferring pups from the holding pen to the trial pen ranged between 1 and 8 min (mean = 3 min). The animals were introduced into the trial pen simultaneously in a standardized manner. Both of the hour-long periods of time spent in the trial pen were recorded (video camera used: Panasonic HDC-TM60 HD 1920 × 1080) from a hide. Forty 3-day trials took place over 16 days of captivity using the two study cohorts, each consisting of an ‘oxytocin manipulation’ and a ‘saline manipulation’ (*n* = 80 manipulation responses). The most extreme reaction possible to another individual in the trial was bites that punctured the skin, and none occurred in any of our trials. To ensure animal welfare was not compromised, all trials were observed by a researcher who could intervene and separate subjects if necessary. This was not required during the study.

### (g) Behavioural observations and decoding

Real-time video footage was decoded after all trials were completed using an ethogram from Robinson *et al*. [14] to produce four metrics for analysis: frequencies of each behaviour type of interest—approach behaviours (‘affiliation’), olfactory and visual investigative behaviours (‘checks’) or aggressive interactions between the trial animals—and the cumulative time in seconds spent within a threshold distance of one body length of each other (‘proximity’). All distances were estimated visually in multiples of weaned pup length, which equates to approximately 1 m.

One investigator (K.J.R.) decoded all the videos for this study, and their error rate was assessed by decoding six different videos twice. The standard error for tallied frequencies of behaviours across the six videos ranged from 0 to 2 per video and the standard error for cumulative time spent within 1 body length in trials ranged from 1 to 31 s.

### (h) Statistical analysis

All analysis was performed using the statistical package R v. 2.15.0 [21]. Significance was defined as *p* ≤ 0.05 for this study.

### (i) Behavioural frequency analysis

Four generalized additive mixed models (GAMMs) [24] were used to investigate the following response variables: (i) the frequency of affiliation, investigative and aggressive behaviours; (ii) the total cumulative number of seconds animals spent within one body length of each other (‘proximity’). Biologically plausible predictor variables considered for inclusion in these models were the sex of the focal individual, the time spent in captivity in days, the type of manipulation given to individuals in a trial (oxytocin or saline), the meeting the pair were given the manipulation (initial or second) and an interaction term between manipulation type and meeting. The identities of both individuals in the trials were fitted as two random effect smooths (focal and response animal) [25] to control for pseudo-replication in the dataset due to use of the same individuals in multiple trials and to control for consistent individual differences in behaviour [23,26]. The smoothing parameters were set by maximum likelihood to reduce the risk of overfitting associated with other methods [27]. The models of the frequencies of investigative, aggressive and affiliative behaviours within the 1 h trial were fitted with Poisson error distributions with log links using the multiple generalized cross-validation library mgcv [28], while the model of time spent within one body length was fitted with a Gaussian distribution. Each model's goodness of fit was examined by calculating *R*^2^ values, AIC scores, QQ and residual plots.

### (j) Post trial plasma oxytocin analysis

Plasma oxytocin concentrations from the weaned pups used in trials at capture (*n* = 20) were compared with samples collected after a subset of saline (*n* = 8) and oxytocin (*n* = 8) manipulation trials to determine whether the manipulations were successful in raising basal oxytocin concentrations for the hour-long trial and if the injection procedure alone elevated plasma oxytocin concentrations. Oxytocin concentrations were compared using a one-way ANOVA after the data underwent a reciprocal transformation, as the original data were not normally distributed (Shapiro–Wilk test, *p* < 0.001) and did not have homogeneous variance across groups (Levene test, *p* = 0.04). There was no difference between the basal oxytocin levels of the study group and free-roaming weaned pups on the Isle of May in 2011, indicating that we had not sub-sampled an unusual or unrepresentative group from the study (electronic supplementary material, SM3).

## 3. Results

### (a) Behavioural frequencies during initial and second meetings with oxytocin and saline manipulations

**Figure 1.**
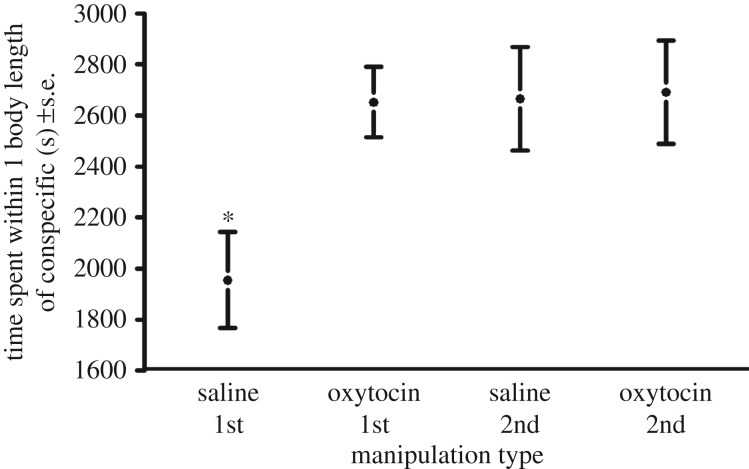
The amount of time spent within one body length of the other trial individual across the initial and second meetings under oxytocin manipulation (*n* = 40) and saline manipulation (*n* = 40) with standard error (SEM) bars. Asterisks denote treatments that are significantly from others. Standard deviations for the groups were: saline 1st: 880.3, oxytocin 1st: 649.1, saline 2nd: 857.1, oxytocin 2nd: 858.0.

**Figure 2.**
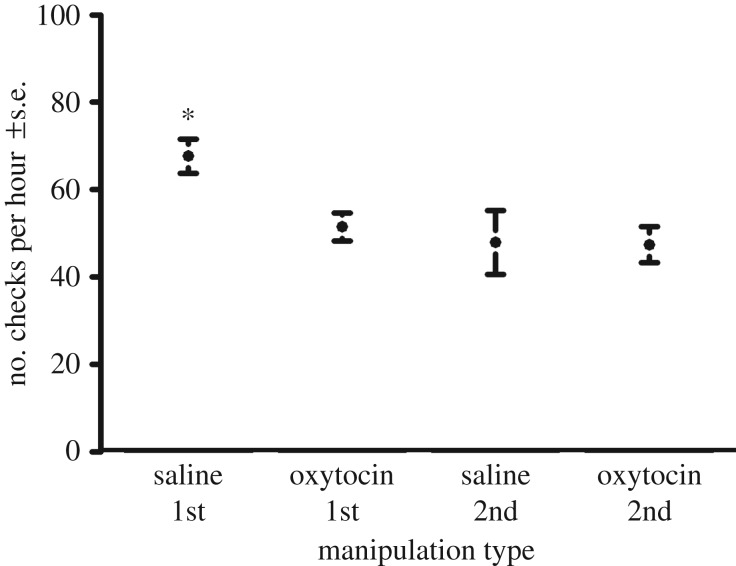
The frequency of investigative behaviours (checks) across the initial and second meetings under oxytocin manipulation (*n* = 40) and saline manipulation (*n* = 40) with SEM bars. Asterisks denote treatments that are significantly from others. Standard deviations for the groups were: saline 1st: 18.7, oxytocin 1st: 15.2, saline 2nd: 30.9, oxytocin 2nd: 17.2.

**Figure 3.**
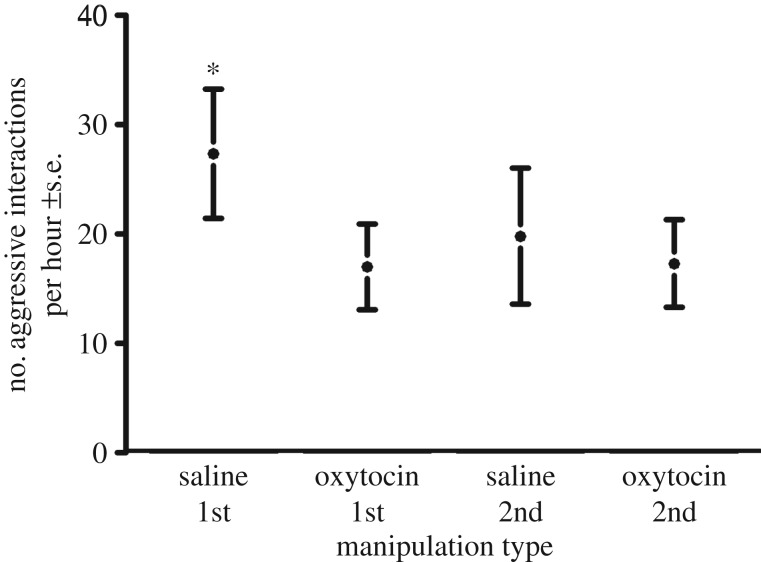
The frequency of aggressive interactions across the initial and second meetings under oxytocin manipulation (*n* = 40) and saline manipulation (*n* = 40) with SEM bars. Asterisks denote treatments that are significantly from others. Standard deviations for the groups were: saline 1st: 27.9, oxytocin 1st: 18.7, saline 2nd: 26.3, oxytocin 2nd: 17.0.

Pups spent significantly more time in close proximity and performed fewer checks and aggressive interactions to the other trial subject during oxytocin manipulation trials (*n* = 40) compared with saline manipulation trials (*n* = 40) (GAMM: *R*^2^ = 0.23, *p* = 0.005, GAMM: *R*^2^ = 0.26, *p* < 0.001, and GAMM: *R*^2^ = 0.61, *p* < 0.001, respectively) (tables 1 and 2, figures 1–3). The number of approaches was not significantly different across manipulation type (GAMM: *R*^2^ = 0.27, *p* = 0.14).

**Table 1.** Mean values (with standard errors) for the frequencies of approaches, checks, aggressive behaviour and the time spent within one body length of the response animal (proximity) for each type of trial.

trial type	meeting type	approaches	checks	aggressive behaviours	time within one body length (proximity) (hours: minutes: seconds)
oxytocin manipulation (*n* = 40)	initial	8.5 (±1.1)	51.4 (±3.2)	17 (±3.9)	00: 44: 12 (±00: 02: 18)
	second	5.8 (±1.2)	47.4 (±1.1)	17.3 (±4)	00: 44: 51 (±00: 03: 22)
saline manipulation (*n* = 40)	initial	7.2 (±1.1)	67.7 (±3.9)	27.3 (±5.9)	00: 32: 35 (±00: 03: 08)
	second	5.3 (±1.4)	48 (±7.3)	19.8 (±6.2)	00: 44: 25 (±00: 03: 22)

**Table 2.** Model output from all GAMMs analysing behavioural responses in recognition trials with their standard errors, estimates and *p*-values. Significant *p*-values (*p* ≤ 0.05) are indicated in italics.

model: response variable	predictor variables	estimate	standard error	*p*-value
approaches	manipulation type (saline)	−0.1	0.09	0.1
	meeting type (second)	−0.3	0.1	*<0*.*001*
	sex (male)	0.2	0.2	0.5
	time in captivity	−0.02	0.02	0.5
checks	manipulation type (saline)	0.2	0.03	*<0*.*001*
	meeting type (second)	−0.2	0.03	*<0*.*001*
	sex (male)	−0.01	0.1	0.9
	time in captivity	−0.02	0.007	*0*.*03*
aggressive interactions	manipulation type (saline)	0.3	0.05	*<0*.*001*
	meeting type (second)	0.03	0.06	0.6
	sex (male)	0.2	0.5	0.7
	time in captivity	−0.1	0.02	*<0*.*001*
proximity	manipulation type (saline)	−687.9	240.1	*0*.*005*
	meeting type (second)	−141.5	255.8	0.6
	interaction term (manipulation and meeting type)	717.6	362.2	*0*.*05*
	sex (male)	−10.03	199.1	0.9
	time in captivity	77.5	39.6	0.06

No predictor variables were eliminated from the model during the selection process. However, the interaction between manipulation (oxytocin/saline) and meeting type (initial/second) was not significant in the approaches, checks and aggressive behaviour models, and was removed, improving the model fit. The interaction between manipulation and meeting type was significant in the proximity model, with individuals in initial saline manipulation trials spending significantly less time in close proximity to the other trial individual (*p* = 0.05, figure 1). Meeting type was a significant variable in both the checks and approaches models, with significantly fewer checks and approaches behaviours in second meetings (*p* < 0.001 for both). As time in captivity increased, pups showed fewer checks and less aggression (*p* = 0.03 and *p* < 0.001 respectively). Sex was not a significant variable in any model but was retained to improve model fit. The individual identities of the trial animals were significant variables in both the investigative behaviour and aggression model (*p* < 0.001 for both, table 3).

**Table 3.** Random effects model outputs from all GAMMs analysing behavioural responses in recognition trials with their standard deviations and *p*-values. Significant *p*-values (*p* ≤ 0.05) are indicated in italics.

model: response variable	GAMM random effect	standard deviation	*p*-value
approaches	focal individual	0.4	0.2
	response individual	0.4	*0*.*03*
checks	focal individual	0.3	*<0*.*001*
	response individual	0.2	*<0*.*001*
aggressive interactions	focal individual	1.2	*<0*.*001*
	response individual	1.4	*<0*.*001*
proximity	focal individual	196.6	0.9
	response individual	196.6	0.9

### (b) Plasma oxytocin concentrations after oxytocin/saline manipulation trials

Plasma oxytocin concentrations were significantly different across post-trial samples and samples taken at capture to obtain basal values for the trial group (ANOVA: *F*_2,33_ = 40.1, *p* < 0.001). Plasma oxytocin concentrations were significantly elevated one hour post oxytocin manipulation (27.3 ± 8.1 pg ml^−1^) compared with samples taken one hour after saline manipulation (8.6 ± 0.8 pg ml^−1^) and basal concentrations (8.5 ± 0.8 pg ml^−1^, Tukey honest significant difference test, *p* < 0.001 for both). Oxytocin concentrations in plasma samples collected one hour after saline manipulation were not significantly different from basal concentrations (*p* = 0.9).

## 4. Discussion

### (a) Pro-social behaviour changes during oxytocin manipulations in grey seals

The oxytocin manipulation designed and used in this study confirmed that elevated oxytocin concentrations cause an increase in proximity-seeking behaviour in this species, a relationship that has been previously detected in natural contexts [13]. This is the first study to identify causality within a naturally existing correlation between behaviour and oxytocin concentrations in a wild species. Linking correlative and manipulation studies in this way confirms not only that the behaviour is modulated by the hormone, but that the behaviour triggered by a manipulation does actually happen outside of experimental contexts. It has previously been hypothesized that oxytocin is important for proximity-seeking behaviours in human mothers [29] and meerkats given intramuscular oxytocin increased the time they spent with pups [6]. Our findings show conclusively that oxytocin modulates this critical behaviour in both experimental and natural settings. More research is needed to investigate the relationship over time between elevated oxytocin concentrations and proximity-seeking behaviour, and how the two may reinforce each other across various types of bonds. In facilitating proximity-seeking behaviours, oxytocin could function to keep bonded dyads together. Stimuli from familiar conspecifics or dependent infants are known to cause oxytocin release [3,30], which this study indicates will lead to individuals seeking close proximity to the individual providing the stimuli. A positive feedback loop may then promote bonded individuals to stay close together, as the more time they spend in close proximity, the more stimuli they will experience from each other, and the more oxytocin will be released to keep a pair close. Such positive feedback loops have been identified in interspecific social bonds (humans–dogs [3]); therefore it is likely they also exist within bonded conspecifics. Positive feedback loops reinforcing mother–infant bonds have previously been suggested [31,32], and our work provides compelling evidence that oxytocin modulates proximity-seeking behaviour as suggested by endogenous hormone–behaviour correlations detected in mother–pup dyads [13].

The oxytocin manipulations in this study also caused changes in other behaviours that could be interpreted as ‘pro-social’, such as the reduction of aggression towards a novel individual and a reduction of investigative behaviours, which typically only happens in this species once individuals are familiar with each other after cohabiting for several days [14,16]. Unlike the correlations documented between oxytocin and proximity-seeking behaviour, peripheral oxytocin concentrations show no change when individual seals are interacting with a familiar or novel conspecifics [14]. In natural interactions between familiar seals, pro-social changes in behavioural expression happen without any detectable change in oxytocin. Changes in oxytocin concentrations could be occurring within central brain structures, which have not been measured in any seal species to date. However, changes in oxytocin due to interactions between socially bonded, unrelated conspecifics frequently do occur in peripheral substrates [10,11], therefore it seems unlikely seals would be an exception to this. This result urges caution when interpreting manipulation results without knowledge of the endogenous oxytocin system in a species, as it shows that even though the hormone is capable of stimulating a variety of pro-social behaviour changes, in natural contexts the elevation of oxytocin may never actually occur in a particular situation. Therefore, we cannot manipulate oxytocin concentrations in individuals and extrapolate that any behavioural changes that occur also happen outside of experimental conditions, as other physiological mechanisms can be responsible for causing behavioural changes that are outwardly visible.

### (b) Peripheral oxytocin manipulations, dose selection and unexpected, persistent social behaviour changes in trial subjects

This study demonstrated that peripheral routes of oxytocin manipulation can be used to stimulate pro-social behaviour changes, despite controversy over their ability to reach central brain structures [15,33] and studies that have encountered difficulties using peripheral injections to cause behavioural changes [34]. Our dose was also able to generate oxytocin concentrations in study individuals one hour post-injection that are comparable to endogenous oxytocin concentrations in this species [13] despite the rapid clearance rate of oxytocin from circulation [12].

While our study shows that IV oxytocin manipulations are effective, it also demonstrates that, even when using the lowest dose possible, it is sufficient to cause unexpected, persistent changes to social behaviour. In seal pairs given the oxytocin manipulation first, the changes in their proximity-seeking, investigative and aggressive behaviours persisted into the second meeting, where they were given saline manipulations. Seals under no manipulation in repeated paired pen trials show no behavioural changes across the two trials, as one hour of cohabitation is not enough time to cause the familiarity needed to reduce aggression and investigative behaviours naturally [16]. Additionally, the same behavioural metrics as those analysed in this study were measured in the repeated paired pen trials using no manipulations, and data from the initial saline trial is comparable with the behaviours recorded when no manipulations are given [16]. Therefore, the changes in these second meetings must be due to the oxytocin manipulation given 2 days earlier, an highly unexpected result given that the dose given would have long been cleared out of circulation [12]. It has been previously shown that oxytocin manipulations may cause persistent social memories to form in trial individuals. Oxytocin injections into central brain regions in mice enable social memory acquisition [35], stimulate the formation of social bonds and preferences in voles [36], increase retention of social memory in rats [37], and in humans have been shown to accelerate the process of social memory formation [38,39] and to increase the retention of social memories [40]. IV injections of oxytocin have additionally been found to produce behavioural changes that persisted across at least 16 days between trials in humans [41]. It is therefore clear that when designing oxytocin manipulations, such long-term impacts on social behaviour must be considered, even in species that demonstrate only limited pro-social behaviour such as seals. With the increasing popularity of human medical trials to use oxytocin to treat psychological conditions [42,43], such unexpected, long-term impacts on social bonding and behaviour must be carefully studied and, if they are not desirable, minimized.

### (c) Conclusion

Oxytocin manipulations have been subject to intense interest in the last decade due to the widespread occurrence of oxytocin in mammals, its far-reaching effects on behaviour (including parental and social bonds) and the potential to use the hormone to treat human psychological conditions. Despite this, no study has confirmed that behavioural changes that occur in laboratory manipulations also occur in natural environments, and many administer doses of oxytocin far exceeding endogenous levels. We show that manipulation studies are possible in wild populations, and that manipulations are most powerful after prior work to identify naturally existing hormone–behaviour relationships. Only then can researchers distinguish between post-manipulation behavioural changes that could occur naturally and ones that could never happen without human intervention. IV manipulations are a viable option for studying the oxytocin system in natural environments, and more research is needed to increase our understanding of this hormone in contexts outside of laboratory studies, which frequently do not replicate all aspects of true biological systems. Finally, the IV oxytocin manipulations used in this study show the hormone is responsible for causing proximity-seeking behaviour in seals, confirming the causal nature of an oxytocin–behaviour relationship that has previously been identified in wild individuals for the first time.

## Supplementary Material

SM 1

## Supplementary Material

SM 2

## Supplementary Material

SM 3

## Supplementary Material

SM 4

## Data accessibility

All relevant data are within the paper and its electronic supplementary material files.
