# Supplementary material for: Positive social behaviours are induced and retained after oxytocin manipulations mimicking endogenous concentrations in a wild mammal
Source: Proc Biol Sci. 2017 May 24;284(1855):20170554. doi: 10.1098/rspb.2017.0554 (PMC5454273; doi:10.1098/rspb.2017.0554)
Supplement: SM 1 [file rspb20170554supp1.doc]

SM 1. Examples of species which have been subjected to oxytocin manipulation experiments and the methodology and outcomes documented.

| Species used | Captive, domestic, laboratory or wild | Manipulation method used | Experimental outcomes | Reference examples |
| --- | --- | --- | --- | --- |
| Bats, vampire (Desmodus rotundus) | Captive | Intranasal | Increases social grooming and food sharing. | [1] |
| Dogs (*Canis lupus familiaris)* | Domestic | Intranasal | Promotes positive social behaviours towards conspecifics and humans. | [2-3] |
| Humans (*Homo sapiens)* | NA | Intranasal^1, 2, 4, 5^  Four hour intravenous infusions^3^ | Mediates stress responses^1^.  Improves ability to infer mental states^2^.  Improves ability to assign emotional significance to speech intonation in autistic patients^3^.  Facilitates approach and bonding behaviours^4^.  Enhances emotional empathy and socially reinforced learning^5^. | ^1^[4]  ^2^[5]  ^3^[6]  ^4^[7]  ^5^[8] |
| Macaques, Rhesus (Macaca mulatta) | Captive | Intranasal | Attenuates attention to negative facial expressions^6^.  Amplifies social motivation and relaxes social vigilance^7^.  Generates elevation of oxytocin in cerebral spinal fluid^8^.  Increases positive social behaviour in newborns^9^.  Increases attention to facial expressions but affect attenuates with dose^10^ | ^6^[9]  ^7^Reviewed in [10]  ^8^[11]  ^9^[12]  ^10^[13] |
| Marmosets (Callithrix penicillata and Callithrix jacchus) | Captive | Intranasal | Increases proximity seeking with bond partner^11^.  Facilitates fidelity in long term partnerships^12^.  Reduces prosocial behaviour towards strangers^13^. | ^11^[14]  ^12^[15-16]  ^13^[17] |
| Meerkat (Suricata suricatta) | Wild | Intramuscular | Elevates cooperative behaviours such as guarding, pup feeding, associating with pups and reduces aggression. | [18] |
| Mice (*Mus musculus*) | Laboratory | Intracerebro-ventricular injection | Inhibits infanticide^14^.  Enables social recognition^15^. | ^14^[19]  ^15^[20] |
| Naked mole rat (Heterocephalus glaber) | Captive | Intraperitoneal injections | Mediates social affiliation by increasing huddling, proximity seeking and investigation of familiar conspecifics. | [21] |
| Rats (*Rattus norvegicus*) | Laboratory | Subcutaneous injection^14^.  Injection into the olfactory bulb^15^ | Facilitates social recognition^16, 17^. | ^16^[22]  ^17^[23] |
| Seals, Grey (*Halichoerus grypus*) and Harbour (*Phoca vitulina*) | Wild | Intravenous injection | Determined plasma clearance rate^18^.  Increases proximity seeking, reduces aggression and investigation of conspecifics^19^. | ^18^[24]  ^19^Robinson *et al.* – findings from this study. |
| Sheep (*Ovis aries*) | Domestic | Intracerebro-ventricular injection | Stimulates maternal behaviour in ewes. | [25-26] |
| Voles, prairie (Microtus ochrogaster) | Laboratory | Intracerebro-ventricular injection^18^.  Intraperitoneal injections^19^.  Subcutaneous injection^20^. | Increases partner preference and contact^20^.  Impacts approach, pup retrieval and time in contact with partner (results dependant on dose given) ^21^.  Reduces negative behavioural consequences of long-term isolation^22^ | ^20^[27]  ^21^[28]  ^22^[29] |

**References**

1. Carter GG, Wilkinson GS. 2015 Intranasal oxytocin increases social grooming and food sharing in the common vampire bat *Desmodus rotundus*. *Horm Behav* **75**, 150-153.
2. Romero T, Nagasawa M, Mogi K, Hasegawa T, Kikusui T. 2014 Oxytocin promotes social bonding in dogs. *P Natl Acad Sci* **111**, 9085-9090.
3. Nagasawa M, Shouhei M, Shiori E, Nobuyo O, Mitsuaki O, Yasuo S, Tatsushi O, Kazutaka M, Takefumi K. 2015 Oxytocin-gaze positive loop and the coevolution of human-dog bonds. *Science* **348**, 333-336.
4. Heinrichs M, Baumgartner T, Kirschbaum C, Ehlert U. 2003 Social support and oxytocin interact to suppress cortisol and subjective responses to psychosocial stress. *Biol Psychiat* **54**, 1389-1398.
5. Domes G, Heinrichs M, Michel A, Berger C, Herpertz SC. 2007 Oxytocin improves “mind-reading” in humans. *Biol Psychiat* **61**, 731-733.
6. Hollander E, Bartz J, Chaplin W, Phillips A, Sumner J, Soorya L, Anagnostou E, Wasserman S. 2007 Oxytocin increases retention of social cognition in autism. *Biol Psychiat* **61**, 498-503.
7. Ditzen B, Schaer M, Gabriel B, Bodenmann G, Ehlert U, Heinrichs M. 2009 Intranasal oxytocin increases positive communication and reduces cortisol levels during couple conflict. *Biol Psychiat*, **65**, 728-731.
8. Hurlemann R, Patin A, Onur OA, Cohen MX, Baumgartner T, Metzler S, Dziobek I, Gallinat J, Wagner M, Maier W, Kendrick KM. 2010 Oxytocin enhances amygdala-dependent, socially reinforced learning and emotional empathy in humans.  *J Neurosci* **30**, 4999-5007.
9. Parr LA, Modi M, Siebert E, Young LJ. 2013 Intranasal oxytocin selectively attenuates rhesus monkeys’ attention to negative facial expressions. *Psychoneuroendocrinol* **38**, 1748-1756.
10. Chang SW, Platt ML. 2014 Oxytocin and social cognition in rhesus macaques: Implications for understanding and treating human psychopathology. *Brain Res* **1580**, 57-68.
11. Dal Monte O, Noble PL, Turchi J, Cummins A, Averbeck BB. 2014 CSF and blood oxytocin concentration changes following intranasal delivery in macaque. *PloS one* **9**, e103677.
12. Simpson EA, Sclafani V, Paukner A, Hamel AF, Novak MA, Meyer JS, Suomi SJ, Ferrari PF. 2014 Inhaled oxytocin increases positive social behaviors in newborn macaques. *P Natl Acad Sci* **111**, 6922-6927.
13. Parr LA, Brooks JM, Jonesteller T, Moss S, Jordano JO, Heitz TR. 2016 Effects of chronic oxytocin on attention to dynamic facial expressions in infant macaques. *Psychoneuroendocrinol* **74**, 149-157.
14. Smith AS, Ågmo A, Birnie AK, French JA. 2010 Manipulation of the oxytocin system alters social behavior and attraction in pair-bonding primates, *Callithrix penicillata*. *Horm Behav* **57**, 255-262.
15. Cavanaugh J, Mustoe AC, Taylor JH, French JA. 2014 Oxytocin facilitates fidelity in well-established marmoset pairs by reducing sociosexual behavior toward opposite-sex strangers. *Psychoneuroendocrinol* **49**, 1-10.
16. Cavanaugh J, Huffman MC, Harnisch AM, French JA. 2015 Marmosets treated with oxytocin are more socially attractive to their long-term mate. *Front Behav Neurosci* **9**, 251.
17. Mustoe AC, Cavanaugh J, Harnisch AM, Thompson BE, French JA. 2015 Do marmosets care to share? Oxytocin treatment reduces prosocial behavior toward strangers. *Horm Behav* **71**, 83-90.
18. Madden JR, Clutton-Brock TH. 2010 Experimental peripheral administration of oxytocin elevates a suite of cooperative behaviours in a wild social mammal. *P Roy Soc Lond B Bio*, DOI:10.1098/rspb.2010.1675.
19. McCarthy MM. 1990 Oxytocin inhibits infanticide in female house mice (*Mus domesticus*). *Horm Behav* **24**, 365-375.
20. Ferguson JN, Aldag JM, Insel TR, Young LJ. 2001 Oxytocin in the medial amygdala is essential for social recognition in the mouse. *J Neurosci* **21**, 8278-8285.
21. Mooney SJ, Douglas NR, Holmes MM. 2014 Peripheral administration of oxytocin increases social affiliation in the naked mole-rat (*Heterocephalus glaber*). *Horm Behav* **65**, 380-385.
22. Popik P, Vetulani J, Van Ree JM. 1992 Low doses of oxytocin facilitate social recognition in rats. *Psychopharmacology* **106**, 71-74.
23. Dluzen DE, Muraoka S, Engelmann M, Landgraf R. 1998 The effects of infusion of arginine vasopressin, oxytocin, or their antagonists into the olfactory bulb upon social recognition responses in male rats. *Peptides* **19**, 999-1005.
24. Robinson KJ, Hazon N, Lonergan M, Pomeroy PP. 2014 Validation of an enzyme-linked immunoassay (ELISA) for plasma oxytocin in a novel mammal species reveals potential errors induced by sampling procedure. *J Neurosci Meth,* **226**, 73-79*.*
25. Kendrick KM, Keverne EB, Baldwin BA. 1987 Intracerebroventricular oxytocin stimulates maternal behaviour in the sheep. *Neuroendocrinol* **46**, 56-61.
26. Da Costa AP, Guevara‐Guzman RG, Ohkura S, Goode JA, Kendrick KM. 1996 The role of oxytocin release in the paraventricular nucleus in the control of maternal behaviour in the sheep. *J Neuroendocrinol* **8**, 163-177.
27. Cho MM, DeVries AC, Williams JR, Carter CS. 1999 The effects of oxytocin and vasopressin on partner preferences in male and female prairie voles (*Microtus ochrogaster*). *Behav Neurosci* **113**, 1071.
28. Bales KL, van Westerhuyzen JA, Lewis-Reese AD, Grotte ND, Lanter JA, Carter CS. 2007 Oxytocin has dose-dependent developmental effects on pair-bonding and alloparental care in female prairie voles. *Horm Behav* **52**, 274-279.
29. Grippo AJ, Trahanas DM, Zimmerman RR, Porges SW, Carter CS. 2009 Oxytocin protects against negative behavioral and autonomic consequences of long-term social isolation. *Psychoneuroendocrinol* **34**, 1542-1553.
