# Supplementary material for: Positive social behaviours are induced and retained after oxytocin manipulations mimicking endogenous concentrations in a wild mammal
Source: Proc Biol Sci. 2017 May 24;284(1855):20170554. doi: 10.1098/rspb.2017.0554 (PMC5454273; doi:10.1098/rspb.2017.0554)
Supplement: SM 2 [file rspb20170554supp2.doc]

SM 2. Dose development for IV oxytocin manipulations in phocid seals.

There is currently little consensus on what constitutes suitable dosages for oxytocin manipulations [1], with many studies to date administering doses that are much higher than physiologically relevant concentrations [2]. Increasing doses of centrally or periperhally administered oxytocin has been shown to attenuate behavioural responses to the hormone (rats: [3], voles: [4], humans: [5], macaques: [6]), attributed to oxytocin receptors desensitising during prolonged elevation [7].

We tested three potential IV oxytocin doses prior to the manipulation trials described in the main text for use in phocid manipulation studies. Dose calculation and peripheral administration were chosen based on the information from previous studies using peripheral oxytocin manipulations to significantly affect behaviour in study animals.

*Ethical Standards*

All procedures involving animals in this study were performed under UK Home Office project licence #60/4009 and conformed to the UK Animals (Scientific Procedures) Act, 1986. The research was approved ethically by the University of St Andrews Animal Welfare and Ethics Committee.

*Study Animals*

One captive harbour seal (*Phoca vitulina*) (individual V) held at the Sea Mammal Research Unit, University of St Andrews (SMRU) was initially utilised to test several potential intravenous (IV) oxytocin doses for phocids. Dose trials were conducted from the 25^th^ June 2011 until the 18^th^ August 2011. Individual V was a two year old male harbour seal who was brought into the captive facility on the 1^st^ October 2010 and was returned to the wild on the 29^th^ October 2011. To document basal plasma oxytocin concentrations in harbour seals to provide a baseline for comparison of post-manipulation plasma samples, plasma samples from 16 wild harbour seals of both sexes (four female, 12 male) from the Shetland Islands were analysed. Samples were collected from the 15^th^ August 2010 until the 20^th^ August 2010.

*Dose Selection*

Several different doses were tested on individual V initially to determine the IV dose most likely to succeed in maintaining elevated plasma oxytocin concentrations for an hour. Trial doses were based on the intramuscular (IM) dose used successfully to cause behavioural changes in free ranging wild meerkats [8]. For all manipulations, we used the same commercially available oxytocin solution as used in the meerkat study (10iu/ml or 0.18mg/ml Oxytocin-S, Intervet UK Ltd). The dose used for meerkats (0.01ml/100g, equal to 18μg/kg) was converted to an IV dose based on the instructions with the oxytocin solution (Intervet UK Ltd), resulting in a 4.5 μg/kg dose. As the blood volume of a phocid seal can be estimated as a percentage of its body mass [9, reviewed in 10], an estimate of the maximal oxytocin concentration in the plasma of an individual post-injection could be calculated to determine how close the concentration was to naturally observed peaks in plasma oxytocin. As these spikes would have been more than a thousand times higher than any naturally occurring oxytocin peak reported in phocid seals [11], the dose was reduced to approximately one tenth of that used in the meerkat experiment. This dose of 0.41μg/kg would still hypothetically generate an initial spike much higher than any recorded natural plasma peak in oxytocin in seals, but was used as a starting point as it was uncertain how high doses needed to be to remain elevated for one hour. Trials testing lower IV doses were also conducted to attempt to find the lowest dose that would still elevate plasma oxytocin concentrations for an hour. The two additional doses included does of approximately half (0.23μg/kg) and one quarter (0.13μg/kg) of the initial 0.41μg/kg dose.

*Initial Dose Testing*

To test if the proposed doses successfully raised plasma oxytocin concentrations for one hour, individual V received a randomised IV injection of one of the oxytocin doses into the extradural vein. Individual V was manually restrained for the injection and then placed in a circular holding pen alone (5m diameter). When one hour had elapsed from the injection, a plasma sample was taken from the extradural vein using manual restraint. Only one dose trial occurred per day to ensure that plasma concentrations returned to basal before another trail occurred. Additionally, one plasma sample was taken prior to the dose trials to obtain a basal oxytocin concentration for individual V. A trial injecting saline solution was also conducted on a separate day to determine if the capture, manual restraint and injection process alone effected plasma oxytocin concentrations.

Oxytocin concentrations were detected in post trial samples and in the basal sample set as described in the main text. Of the three doses tested, all except the lowest, 0.13μg/kg dose succeeded in elevating plasma oxytocin concentrations above basal (6.6 ±0.8pg/ml, n=17) one hour after the injection (post 0. 0.13μg/kg: 7.3pg/ml, post 0.23μg/kg: 12.6pg/ml, post 0.41μg/kg: 25.3pg/ml). Plasma oxytocin concentrations in the basal group were comparable to concentrations one hour post IV saline injection (6.4pg/ml). Plasma oxytocin concentrations detected 1 hour post injection for the 0.23μg/kg and 0.41μg/kg doses were both within the range of naturally occurring plasma oxytocin concentrations in phocid seals [11].

*Dose Selection*

Of the doses tested above, the 0.41μg/kg dose was selected to be used in manipulation experiments outside of laboratory conditions. The 0.41μg/kg dose was selected out of the two tested doses that did raise plasma oxytocin concentrations above basal for one hour, based on the hypothetical concentrations of oxytocin that would cross the blood brain barrier (BBB) within 10 minutes of injection. Mens *et al.* [12] showed that 0.002% of an oxytocin dose administered IV or subcutaneously to freely moving rats was detectable in the cerebrospinal fluid (CSF) within ten minutes of the injection. If this figure is used in conjunction with the doses developed in this study and the typical mass of grey seal pups at weaning (40kg, [13]), the approximate amount of oxytocin crossing into the CSF of seals ten minutes after being given this dose can be calculated (360pg for the 0.41μg/kg dose). Behavioural manipulations using central injections have been conducted with doses as low as a single 500pg injection [14], therefore the 0.41μg/kg dose was selected for further investigation as it was the lowest dose that persisted for an hour that hypothetically would generate an amount of oxytocin crossing the BBB similar to amounts administered centrally in successful behaviour manipulation trials [14].

The three doses tested for use prior this study were all much lower than those used in the majority of peripheral manipulation experiments previously, even when taking into account conversions from IV to other peripheral routes of injection (15.2μg per individual mouse [15], 0.1-20μg per individual vole [16], 2000μg/kg, 4000μg/kg and 8000μg/kg [4], 20μg per individual vole [17], 18μg/kg [8], 1000-10,000μg/kg; [18]). Therefore there is great potential for refinement of doses used in future manipulation studies, which would benefit the individuals used in the studies and the scientific outcomes of the research by more closely replicating natural systems.

*Discussion of oxytocin dose selection and dosing methodology.*

The three doses tested here, including the one successfully used for the manipulation trials to alter behaviour described in the main text of the publication, were all much lower than those used in the majority of peripheral oxytocin manipulation experiments previously. It is clear that peripheral oxytocin manipulations will only be successful if careful consideration and, if possible, pilot studies exploring the viability and suitability of doses take place. Unless efforts are made to generate biologically relevant concentrations, which typically can only be achieved by determining the lowest possible functional dose for use in peripheral manipulation studies, there is a risk of generating supraphysiological concentrations that can have unintended negative side effects on trial individuals [2] as well as causing behavioural changes that are not a true representations of the natural relationship between oxytocin and behaviour. We have shown that with diligent development of dose prior to manipulation, it is possible to use IV oxytocin injections low enough to represent endogenous concentrations during hour long trials while still being high enough to permeate the BBB so that behavioural changes could take place. Striking this balance between supraphysiological concentrations and doses too low to cause significant behavioural effects is therefore a crucial, and often overlooked, aspect of oxytocin manipulation studies to date. The variability in oxytocin manipulation studies means that there is still a need for research into ways to administer the hormone, with methods that deliver a consistent dose reliably and across many individuals and experimental conditions, especially if the hormone is to be used in medical treatments for humans.

Injecting oxytocin doses via peripheral routes may prove a viable alternative to central injections or intranasal sprays, providing a compromise between invasiveness and reliability of dosing subjects. While endogenous peripheral oxytocin is unable to cross the BBB, appropriate doses of exogenous oxytocin cause biologically significant amounts of the neuropeptide to reach central brain structures (rats [19]), as seen in this study. Currently, intranasal sprays are popular for non-human primate [20-22] and human (23-26] manipulation experiments. While these are non-invasive, intranasal sprays have their drawbacks and their widespread use in behavioural manipulation trials has been criticised [2]. Intranasal methodologies may not consistently administer oxytocin doses accurately [21, 22], may not be possible for some mammalian species depending on their nasal structure, or may have to be significantly modified to allow successful administration of the oxytocin dose [19]. Our success with using IV injections of oxytocin in a wild mammal species show that it is possible to develop a dose of this neuropeptide that persists across an hour, which can be administered rapidly and consistently to all trial subjects and therefore has the potential to be used in behavioural manipulation trials on other wild animal subjects.

References

1. Churchland PS, Winkielman P. 2012 Modulating social behavior with oxytocin: how does it work? What does it mean? *Horm Behav* **61**, 392-399.
2. Leng G, Ludwig M. 2016 Intranasal oxytocin: myths and delusions. *Biol Psychiat* **79**, 243-250.
3. Popik P, Vetulani J, Van Ree JM. 1992 Low doses of oxytocin facilitate social recognition in rats. *Psychopharmacol* **106**, 71-74.
4. Bales KL, van Westerhuyzen JA, Lewis-Reese AD, Grotte ND, Lanter JA, Carter CS. 2007 Oxytocin has dose-dependent developmental effects on pair-bonding and alloparental care in female prairie voles. *Horm Behav* **52**, 274-279.
5. Hurlemann R, Patin A, Onur OA, Cohen MX, Baumgartner T, Metzler S, Dziobek I, Gallinat J, Wagner M, Maier W, Kendrick KM. 2010 Oxytocin enhances amygdala-dependent, socially reinforced learning and emotional empathy in humans.  *J Neurosci* **30**, 4999-5007.
6. Parr LA, Brooks JM, Jonesteller T, Moss S, Jordano JO, Heitz TR. 2016 Effects of chronic oxytocin on attention to dynamic facial expressions in infant macaques. *Psychoneuroendocrinol* **74**, 149-157.
7. Phaneuf S, Linares BR, TambyRaja RL, MacKenzie IZ, Bernal AL. 2000 Loss of myometrial oxytocin receptors during oxytocin-induced and oxytocin-augmented labour. *J Reprod Fertil* **120**, 91-97.
8. Madden JR, Clutton-Brock TH. 2010 Experimental peripheral administration of oxytocin elevates a suite of cooperative behaviours in a wild social mammal. *P Roy Soc Lond B Bio*, DOI:10.1098/rspb.2010.1675.
9. Castellini MA, Somero GN. 1981 Buffering capacity of vertebrate muscle: correlations with potentials for anaerobic function. *J Comp Physiol* **143**, 191-198.
10. Kooymann GL. 1989 *Diverse Divers, Physiology and Behaviour.* Berlin: Springer-Verlag.
11. Robinson KJ, Twiss SD, Hazon N, Pomeroy PP. 2015 Maternal oxytocin is linked to close mother-infant proximity in grey seals (*Halichoerus grypus*). *PloS one* **10**, e0144577.
12. Mens WB, Witter A, van Wimersma Greidanus TB. 1983 Penetration of neurohypophyseal hormones from plasma into cerebrospinal fluid (CSF): half-times of disappearance of these neuropeptides from CSF. *Brain Res* **262**, 143-149.
13. Hall AJ, McConnell BJ, Barker RJ. 2001 Factors affecting first-year survival in grey seals and their implications for life history strategy. *J Anim Ecol* **70**, 138-149.
14. Dluzen DE, Muraoka S, Engelmann M, Landgraf R. 1998 The effects of infusion of arginine vasopressin, oxytocin, or their antagonists into the olfactory bulb upon social recognition responses in male rats. *Peptides* **19**, 999-1005.
15. McCarthy MM. 1990 Oxytocin inhibits infanticide in female house mice (*Mus domesticus*). *Horm Behav* **24**, 365-375.
16. Cushing BS, Carter CS. 2000 Peripheral pulses of oxytocin increase partner preferences in female, but not male, prairie voles. *Horm Behav* **37**, 49-56.
17. Grippo AJ, Trahanas DM, Zimmerman RR, Porges SW, Carter CS. 2009 Oxytocin protects against negative behavioral and autonomic consequences of long-term social isolation. *Psychoneuroendocrinol* **34**, 1542-1553.
18. Mooney SJ, Douglas NR, Holmes MM. 2014 Peripheral administration of oxytocin increases social affiliation in the naked mole-rat (*Heterocephalus glaber*). *Horm Behav* **65**, 380-385.
19. Neumann ID, Maloumby R, Beiderbeck DI, Lukas M, Landgraf R. 2013 Increased brain and plasma oxytocin after nasal and peripheral administration in rats and mice. *Psychoneuroendocrino* **38**, 1985-1993.
20. Smith AS, Ågmo A, Birnie AK, French JA. 2010 Manipulation of the oxytocin system alters social behavior and attraction in pair-bonding primates, *Callithrix penicillata*. *Horm Behav* **57**, 255-262.
21. Dal Monte O, Noble PL, Turchi J, Cummins A, Averbeck BB. 2014 CSF and blood oxytocin concentration changes following intranasal delivery in macaque. *PloS one* **9**, e103677.
22. Modi ME, Connor-Stroud F, Landgraf R, Young LJ, Parr LA. 2014 Aerosolized oxytocin increases cerebrospinal fluid oxytocin in rhesus macaques. *Psychoneuroendocrinol* ***45***, 49-57.
23. Rimmele U, Hediger K, Heinrichs M, Klaver P. 2009 Oxytocin makes a face in memory familiar. *J Neurosci* **29**, 38-42.
24. Heinrichs M, Baumgartner T, Kirschbaum C, Ehlert U. 2003 Social support and oxytocin interact to suppress cortisol and subjective responses to psychosocial stress. *Biol Psychiat* **54**, 1389-1398.
25. Kosfeld M, Heinrichs M, Zak PJ, Fischbacher U, Fehr E. 2005 Oxytocin increases trust in humans. *Nature* **435**, 673-676.
26. Ditzen B, Schaer M, Gabriel B, Bodenmann G, Ehlert U, Heinrichs M. 2009 Intranasal oxytocin increases positive communication and reduces cortisol levels during couple conflict. *Biol Psychiat* **65**, 728-731.
