# Supplementary material for: Positive social behaviours are induced and retained after oxytocin manipulations mimicking endogenous concentrations in a wild mammal
Source: Proc Biol Sci. 2017 May 24;284(1855):20170554. doi: 10.1098/rspb.2017.0554 (PMC5454273; doi:10.1098/rspb.2017.0554)
Supplement: SM 3 [file rspb20170554supp3.doc]

SM 3. Basal plasma oxytocin detection across different grey seal colonies.

*Ethical Standards*

All procedures involving animals in this study were performed under UK Home Office project licence #60/4009 and conformed to the UK Animals (Scientific Procedures) Act, 1986. The research was approved ethically by the University of St Andrews Animal Welfare and Ethics Committee.

*Plasma Sampling and Analysis*

Basal plasma oxytocin concentrations were calculated for 40 free-roaming weaned pups from the Isle of May colony in 2010 (n=12) and 2011 (n=28) and for 36 free-roaming weaned pups from North Rona in 2009 (n=8), 2010 (n=3) and 2011 (n=15). These were compared using a one-way ANOVA to the study group’s plasma oxytocin concentrations at capture to check that the trial subjects were not unusual. The data were analysed after a natural log transformation as the original data were not normally distributed (Shapiro Wilk test, p<0.01).

Basal plasma oxytocin concentrations differed across groups of weaned pups from different breeding colonies and different years (ANOVA: F_(5, 90)_ = 8.4, p<0.001, Table SI 3.1). No statistically significant differences were detected between the 2011 trial group and the free roaming individuals from the Isle of May in 2011 (8.6 ±0.85 pg/ml and 9.9 ±1 pg/ml respectively, Tukey honest significant difference test, p=0.5). However there were significant differences between some cohorts sampled in different colonies during different years. The cohorts sampled on the Isle of May in 2010 (13.6 ±1.3 pg/ml) and North Rona in 2011 (12.4 ±1.2 pg/ml) were significantly higher than all other cohorts (p<0.05 for all comparisons) but were not different from each other (p=0.9). There were no significant differences between any other cohorts (p values range between 0.2 – 0.9).

Table SM 3.1. Mean plasma oxytocin concentrations (with standard errors) in the trial group at capture and in free-roaming weaned pups on two colonies across three years of sampling effort.

| **Cohort** | **North Rona 2009** | **North Rona 2010** | **North Rona 2011** | **Isle of May 2010** | **Isle of May 2011** | **Trial Group** |
| --- | --- | --- | --- | --- | --- | --- |
| Plasma oxytocin concentrations (pg/ml) | 7.8 (±0.8) | 9.1 (±0.95) | 12.4 (±1.2) | 13.6 (±1.3) | 9.9 (±1) | 8.6 (±0.85) |

There was no difference between the basal oxytocin levels of the study group and free-roaming weaned pups on the Isle of May in 2011, indicating that we had not sub-sampled an unusual and unrepresentative group from the study. However, there were differences between some basal datasets from different years of sampling and different colonies. While it is possible these are true differences in basal oxytocin concentration across different colonies and years, we hypothesis that oxytocin concentrations in weaned individuals are dependant on the time since weaning event and the last contact with the mother. Thus the differences detected here are most likely due to accidental sampling of subsets of weaners of different ages. This is supported by comparing the basal oxytocin concentrations in a study group of weaned pups from the Isle of May from another pen trial study in 2010 [1] to the current study in 2011. Individuals in the 2010 trails were penned within a shorter time period since weaning (mean=2 days post wean, SD = 1.1 days) compared to the 2011 group (mean=3 days post wean, SD = 1.5 days), and the plasma oxytocin concentrations were significantly different to each other with the 2010 study group (mean = 14.2 ±1.61 pg/ml, [1]) having higher concentrations than the current study group in 2011 (mean = 8.6 ±0.85 pg/ml).

References

1. Robinson KJ, Twiss SD, Hazon N, Moss S, Lonergan M, Pomeroy PP. 2015 Conspecific recognition and aggression reduction to familiars in newly weaned, socially plastic mammals. *Behav Ecol Sociobiol*, **69**, 1383-1394.
