# Supplementary material for: Positive social behaviours are induced and retained after oxytocin manipulations mimicking endogenous concentrations in a wild mammal
Source: Proc Biol Sci. 2017 May 24;284(1855):20170554. doi: 10.1098/rspb.2017.0554 (PMC5454273; doi:10.1098/rspb.2017.0554)
Supplement: SM 4 [file rspb20170554supp4.doc]

SM 4. Original Data

SM 4.1. Original Data – Behavioural data and associated metadata across oxytocin and saline manipulation trials.

| Identity of focal seal in trial | Identity of other seal in the trial | Sex of focal seal | Number of checks/ trial | Number of aggressive behaviours/ trial | Number of approaches/ trial | Time spent in close proximity (seconds) in trial | Injection given (IV) | Meeting type | Number of days in holding pens |
| --- | --- | --- | --- | --- | --- | --- | --- | --- | --- |
| 1 | E | M | 74 | 39 | 7 | 2242 | Oxytocin | Second | 4 |
| E | 1 | M | 58 | 2 | 10 | 2242 | Oxytocin | Second | 4 |
| 1 | E | M | 71 | 40 | 5 | 2602 | Saline | First | 2 |
| E | 1 | M | 82 | 3 | 14 | 2602 | Saline | First | 2 |
| 3 | A | F | 85 | 81 | 14 | 3142 | Oxytocin | First | 2 |
| A | 3 | F | 64 | 2 | 4 | 3142 | Oxytocin | First | 2 |
| 3 | A | F | 99 | 63 | 27 | 2472 | Saline | Second | 4 |
| A | 3 | F | 100 | 28 | 5 | 2472 | Saline | Second | 4 |
| 4 | C | F | 31 | 16 | 0 | 623 | Oxytocin | Second | 4 |
| C | 4 | F | 28 | 7 | 1 | 623 | Oxytocin | Second | 4 |
| 4 | C | F | 106 | 72 | 5 | 1973 | Saline | First | 2 |
| C | 4 | F | 45 | 10 | 11 | 1973 | Saline | First | 2 |
| 2 | B | M | 47 | 7 | 15 | 2814 | Oxytocin | First | 2 |
| B | 2 | F | 29 | 31 | 3 | 2814 | Oxytocin | First | 2 |
| 2 | B | M | 26 | 13 | 2 | 228 | Saline | Second | 4 |
| B | 2 | F | 28 | 21 | 4 | 228 | Saline | Second | 4 |
| 5 | D | F | 50 | 40 | 4 | 2957 | Oxytocin | Second | 4 |
| D | 5 | M | 54 | 25 | 10 | 2957 | Oxytocin | Second | 4 |
| 5 | D | F | 94 | 2 | 2 | 306 | Saline | First | 2 |
| D | 5 | M | 62 | 35 | 0 | 306 | Saline | First | 2 |
| 5 | B | F | 67 | 5 | 18 | 2610 | Oxytocin | Second | 10 |
| B | 5 | F | 63 | 0 | 9 | 2610 | Oxytocin | Second | 10 |
| 5 | B | F | 44 | 0 | 9 | 1578 | Saline | First | 6 |
| B | 5 | F | 38 | 2 | 4 | 1578 | Saline | First | 6 |
| 1 | D | M | 65 | 25 | 19 | 2233 | Oxytocin | First | 6 |
| D | 1 | M | 74 | 9 | 14 | 2233 | Oxytocin | First | 6 |
| 1 | D | M | 54 | 22 | 9 | 2860 | Saline | Second | 8 |
| D | 1 | M | 48 | 0 | 5 | 2860 | Saline | Second | 8 |
| 2 | A | M | 48 | 38 | 4 | 826 | Saline | First | 6 |
| A | 2 | F | 50 | 2 | 7 | 826 | Saline | First | 6 |
| 4 | E | F | 38 | 26 | 6 | 3205 | Oxytocin | First | 6 |
| E | 4 | M | 36 | 4 | 5 | 3205 | Oxytocin | First | 6 |
| 4 | E | F | 110 | 81 | 1 | 3066 | Saline | Second | 8 |
| E | 4 | M | 88 | 31 | 8 | 3066 | Saline | Second | 8 |
| 3 | C | F | 58 | 9 | 16 | 3229 | Oxytocin | Second | 8 |
| C | 3 | F | 56 | 61 | 5 | 3239 | Oxytocin | Second | 8 |
| 3 | C | F | 68 | 25 | 11 | 2698 | Saline | First | 6 |
| C | 3 | F | 77 | 96 | 3 | 2698 | Saline | First | 6 |
| 5 | A | F | 55 | 15 | 6 | 3097 | Oxytocin | First | 8 |
| A | 5 | F | 45 | 3 | 0 | 3097 | Oxytocin | First | 8 |
| 6 | H | F | 43 | 3 | 12 | 3244 | Oxytocin | First | 2 |
| H | 6 | F | 78 | 41 | 3 | 3244 | Oxytocin | First | 2 |
| 6 | H | F | 42 | 0 | 6 | 3015 | Saline | Second | 4 |
| H | 6 | F | 44 | 11 | 4 | 3015 | Saline | Second | 4 |
| 9 | I | M | 42 | 3 | 4 | 3600 | Oxytocin | Second | 4 |
| I | 9 | M | 37 | 27 | 2 | 3600 | Oxytocin | Second | 4 |
| 9 | I | M | 83 | 8 | 12 | 3078 | Saline | First | 2 |
| I | 9 | M | 79 | 79 | 8 | 3078 | Saline | First | 2 |
| 7 | K | M | 59 | 13 | 10 | 2986 | Oxytocin | First | 2 |
| K | 7 | F | 51 | 13 | 8 | 2986 | Oxytocin | First | 2 |
| 7 | K | M | 44 | 7 | 1 | 3050 | Saline | Second | 4 |
| K | 7 | F | 31 | 1 | 5 | 3050 | Saline | Second | 4 |
| 11 | F | F | 35 | 24 | 3 | 2883 | Oxytocin | Second | 4 |
| F | 11 | M | 31 | 4 | 4 | 2883 | Oxytocin | Second | 4 |
| 11 | F | F | 93 | 17 | 16 | 1453 | Saline | First | 2 |
| F | 11 | M | 75 | 41 | 14 | 1453 | Saline | First | 2 |
| X | G | M | 44 | 0 | 15 | 1120 | Oxytocin | First | 2 |
| G | X | M | 47 | 38 | 4 | 1120 | Oxytocin | First | 2 |
| X | G | M | 20 | 0 | 3 | 2748 | Saline | Second | 4 |
| G | X | M | 11 | 0 | 1 | 2748 | Saline | Second | 4 |
| 9 | G | M | 58 | 0 | 11 | 2530 | Oxytocin | First | 6 |
| G | 9 | M | 22 | 16 | 2 | 2530 | Oxytocin | First | 6 |
| 9 | G | M | 52 | 0 | 11 | 2980 | Saline | Second | 8 |
| G | 9 | M | 31 | 23 | 0 | 2980 | Saline | Second | 8 |
| 6 | I | F | 12 | 3 | 1 | 2640 | Oxytocin | Second | 8 |
| I | 6 | M | 29 | 0 | 4 | 2640 | Oxytocin | Second | 8 |
| 6 | I | F | 62 | 16 | 0 | 2290 | Saline | First | 6 |
| I | 6 | M | 60 | 2 | 15 | 2290 | Saline | First | 6 |
| 11 | K | F | 46 | 18 | 8 | 2918 | Oxytocin | First | 6 |
| K | 11 | F | 53 | 7 | 11 | 2918 | Oxytocin | First | 6 |
| 11 | K | F | 2 | 1 | 0 | 3017 | Saline | Second | 8 |
| K | 11 | F | 12 | 0 | 2 | 3017 | Saline | Second | 8 |
| 7 | H | M | 61 | 24 | 5 | 3427 | Oxytocin | Second | 8 |
| H | 7 | F | 67 | 23 | 2 | 3427 | Oxytocin | Second | 8 |
| 7 | H | M | 50 | 34 | 1 | 2748 | Saline | First | 6 |
| H | 7 | F | 66 | 23 | 3 | 2748 | Saline | First | 6 |
| X | F | M | 47 | 3 | 10 | 1888 | Oxytocin | First | 6 |
| F | X | M | 46 | 19 | 6 | 1888 | Oxytocin | First | 6 |
| X | F | M | 66 | 12 | 10 | 3215 | Saline | Second | 8 |
| F | X | M | 52 | 82 | 2 | 3215 | Saline | Second | 8 |

SM 4.2. Original Data – Plasma oxytocin concentrations at capture from the wild, one hour post IV oxytocin injection and one hour post IV saline injection.

| Sample Type | Oxytocin Concentration (pg/ml) |
| --- | --- |
| capture | 8.899434187 |
| capture | 8.249383756 |
| capture | 10.95524316 |
| capture | 7.378047696 |
| capture | 7.198014331 |
| capture | 8.116561888 |
| capture | 10.02795313 |
| capture | 10.48558377 |
| capture | 13.20369787 |
| capture | 8.825765109 |
| capture | 7.396297781 |
| capture | 7.621838516 |
| capture | 6.940877185 |
| capture | 8.01773055 |
| capture | 6.810665195 |
| capture | 9.411473701 |
| capture | 7.282072628 |
| capture | 11.36779399 |
| capture | 7.513122671 |
| capture | 9.456837046 |
| post oxytocin | 27.79107892 |
| post oxytocin | 20.12079566 |
| post oxytocin | 31.35026914 |
| post oxytocin | 27.04756635 |
| post oxytocin | 29.06362386 |
| post oxytocin | 25.66597785 |
| post oxytocin | 45.13984833 |
| post oxytocin | 23.51245417 |
| post saline | 7.175786778 |
| post saline | 8.96353077 |
| post saline | 11.09171633 |
| post saline | 14.79435888 |
| post saline | 8.602786381 |
| post saline | 9.041192794 |
| post saline | 5.430395115 |
| post saline | 9.208240525 |
